# Computational imaging for rapid detection of grade-I cerebral small vessel disease (cSVD)

**DOI:** 10.1016/j.heliyon.2024.e37743

**Published:** 2024-09-11

**Authors:** Saman Shahid, Aamir Wali, Sadaf Iftikhar, Suneela Shaukat, Shahid Zikria, Jawad Rasheed, Tunc Asuroglu

**Affiliations:** a Department of Sciences & Humanities, National University of Computer & Emerging Sciences (NUCES)-FAST Lahore Campus, Punjab, Pakistan; b Department of Data Sciences, National University of Computer & Emerging Sciences (NUCES)-FAST Lahore Campus, Punjab, Pakistan; c Department of Neurology, King Edward Medical University/Mayo Hospital, Lahore, Punjab, Pakistan; d Department of Radiology, King Edward Medical University/Mayo Hospital, Lahore, Punjab, Pakistan; e Department of Computer Science, Information Technology University (ITU), Lahore, Punjab, Pakistan; f Department of Computer Engineering, Istanbul Sabahattin Zaim University, Istanbul, 34303, Turkey; g Department of Software Engineering, Istanbul Nisantasi University, Istanbul, Turkey; h Deep Learning and Medical Image Analysis Laboratory, Bogazici University, Istanbul, Turkey; i Faculty of Medicine and Health Technology, Tampere University, Tampere, Finland

**Keywords:** Computational imaging, Cerebral small vascular disease (cSVD) grade-1, 3D CNN (convolutional neural network), Magnetic resonance image (MRI), Custom dataset

## Abstract

An early identification and subsequent management of cerebral small vessel disease (cSVD) grade 1 can delay progression into grades II and III. Machine learning algorithms have shown considerable promise in medical image interpretation automation. An experimental cross-sectional study aimed to develop an automated computer-aided diagnostic system based on AI (artificial intelligence) tools to detect grade 1-cSVD with improved accuracy. Patients with Fazekas grade 1 cSVD on Non-Contrast Magnetic Resonance Imaging (MRI) Brain of age >40 years of both genders were included. The dataset was pre-processed to be fed into a 3D convolutional neural network (CNN) model. A 3D stack with the shape (120, 128, 128, 1) containing axial slices from the brain magnetic resonance image was created. The model was created from scratch and contained four convolutional and three fully connected (FC) layers. The dataset was preprocessed by making a 3D stack, and normalizing, resizing, and completing the stack was performed. A 3D-CNN model architecture was designed to train and test preprocessed images. We achieved an accuracy of 93.12 % when 2D axial slices were used. When the 2D slices of a patient were stacked to form a 3D image, an accuracy of 85.71 % was achieved on the test set. Overall, the 3D-CNN model performed very well on the test set. The earliest and the most accurate diagnosis from computational imaging methods can help reduce the huge burden of cSVD and its associated morbidity in the form of vascular dementia.

## 1 Introduction

Cerebral small vessel disease (cSVD) is a condition of cerebral microvessels and is becoming increasingly prevalent across the world. These alterations affect the arterioles, capillaries, and small vessels that supply the brain's white matter and deep structures. Cerebral small vessel disease (cSVD) is a major cause of stroke and dementia. It is responsible for around a quarter of all ischemic strokes and the majority of hemorrhagic strokes. It is the most prevalent cause of vascular dementia and worsens the associated cognitive impairment, accounting for approximately 50 % of dementia globally, resulting in a significant health burden. Cerebrovascular disease is the most prevalent cause of stroke and also the world's second-leading cause of mortality [[1], [2], [3], [4], [5], [6], [7], [8]]. Studies have shown that cSVD is linked to strokes, brain hemorrhages, and other neurological disorders, such as Alzheimer's and Parkinson's disease [2]. The currently available methods for reliably identifying cSVD are neuroimaging, such as MRI and CT scans. Essentially, the established imaging biomarkers of cSVD, such as white matter hyperintensities (WMH), lacunes, small subcortical infarcts (SBI), etc., are detected for the diagnosis of cSVD [2,5,6]. According to the guidelines for reporting vascular alterations on neuroimaging (STRIVE), brain shrinkage is one of the major radiological descriptions in the context of cSVD [9]. Neuronal loss, cortical thinning, a subcortical vascular disease with white matter thinning and contraction, arteriolar sclerosis, venous collagen degeneration, and subsequent neurodegenerative alterations are among the neuropathological causes [10]. Brain shrinkage frequently occurs in conjunction with other symptoms of cSVD, and it is an essential parameter in brain imaging used to quantify a load of vascular injury in the brain [[11], [12], [13]].

Cerebral small vessel disease (cSVD) is linked with a surprising degree of diversity in clinical symptoms — both in type and severity — that cannot be entirely explained by traditional SVD indicators. Conventional MRI does not capture the heterogeneity inherent in SVD lesions that seem identical and displays just the tip of the iceberg of overall SVD-related brain damage. SVD impacts brain tissue beyond the generally recognized localized lesions by triggering a cascade of events that extend from the primary lesion to distant brain locations, likely contributing to clinical outcomes. SVD impairs anatomical and functional network connection, impairing effective communication in brain networks, which is required for functional performance. Brain resilience, which defends against clinical deterioration induced by SVD via reserve and compensating systems, explains the clinical variance seen in individuals with seemingly identical SVD lesion load. The clinical concept that SVD is mostly a subcortical disease with isolated lesions has to be reconsidered [14].

SVD imaging features include ischemic white matter lesions (WML) and lacunar infarcts, both of which have been linked to long-term cognitive deterioration. Individually, the clinical picture of SVD is heterogeneous, and the reasons for poor cognitive prognosis remain unknown. Concomitant regional and widespread brain shrinkage, in addition to vascular disease, may influence clinical outcomes. In people without dementia, brain atrophy, as shown by reduced gray matter and hippocampal volumes and greater CSF volumes, correlates with WML volume. Significant hippocampal neuronal loss has been reported in patients with SVD. Brain shrinkage and WML are both linked to long-term cognitive deterioration in small vessel disease. White matter hyperintensities (WMHs) on T2-weighted imaging, tiny infarcts, macro-hemorrhages, dilated perivascular spaces, microbleeds, and brain atrophy are prominent hallmarks of SVDs apparent on conventional brain magnetic resonance images. WMHs are the most prevalent and generally the first brain tissue alterations to appear. Furthermore, significant population- and patient-based research has demonstrated the clinical relevance of WMHs, particularly concerning cognitive and motor impairments, over the last two decades [[15], [16], [17], [18]] cSVD is an age and risk factor-related disease of the brain's microvascular that can develop into incapacitating conditions such as vascular cognitive impairment (VCI) and lacunar stroke [19]. Lacunar stroke syndromes account for around one-fifth of all symptomatic strokes that are linked to cSVD [[19], [20], [21], [22], [23], [24]].

In the field of medicine and healthcare, computer vision has brought about a significant revolution. A machine trained on a medical image data set can deliver accurate results within seconds, whereas a physician may take some time to reach the same conclusion. Modern healthcare systems rely on computer vision and image processing algorithms as fundamental components. Although neuroimaging approaches for detecting grade-1 cSVD are quick and accurate, they require specialist radiologists who may not be readily available, especially in remote parts of developing nations. Convolutional neural networks (CNN) are widely employed to process medical images. In 2012, AlexNet [25], a convolutional neural network running on GPU, won the ImageNet Large Scale Visual Recognition Challenge. Deep learning has seen increased usage in recent years to tackle medical problems such as recognizing lung complaints, locating brain tumors, and so on [[26], [27], [28], [29], [30], [31], [32], [33]], and such studies have also been shown to help medically. CNNs have been used for the detection of lacunes from MRIs. In a study, CNN was used to segment WMHs (white matter hyperintensities) biomarkers from MR images [2,20,34].

An early and accurate diagnosis of grade 1 cSVD on imaging can help in reducing the huge burden of this disease, which is associated with morbidity in the form of vascular dementia. It will be quite helpful in the early identification and subsequent management of cSVD grade 1 to delay the progression into grades II and III by addressing the risk factors such as diabetes mellitus or hypertension at an earlier stage. This can significantly improve a patient's quality of life. Patients with grade 1-cSVD are often overlooked as they do not seek consultation at an earlier stage. Caregivers of patients with cSVD consult the neuro-physician when it gets too late, and this turns into later forms of the disease. This study aimed to develop an automated computer-aided diagnostic system based on AI tools and decision models that could diagnose grade 1-cSVD at an early stage and with greater accuracy. Early and correct identification of grade 1 cSVD on imaging will assist in reducing the enormous burden of this common entity and its accompanying morbidity in the form of vascular dementia. Data sets were acquired from the Mayo Hospital in Lahore with the permission of a neurophysician. It includes cases of confirmed cSVD grade-1 as well as normal cases. We employed CNN techniques to extract significant features, which we refer to as AI-based markers, to predict grade-1 cSVD automatically.

## 2 Materials & methods

### 2.1 Study design & setting

An experimental cross-sectional study was conducted at the Neurology Department, King Edward Medical University (KEMU), Mayo Hospital, and the National University of Computer and Emerging Sciences (NUCES), Lahore, Pakistan. The study was conducted from February 1, 2023 to December 31, 2023. Prior ethical approval was taken from the hospital. The data was collected with informed consent from the patient. There were 30 patients with grade 1 cSVD (Fazekas grading) and 15 individuals in the control group. STROBE guidelines were followed for the study.

### 2.2 Inclusion and exclusion criteria

Patients who had diabetes as well as hypertensives, having grade 1 cSVD (Fazekas grading) on MRI Brain having age >40 years of both genders, were included. Controls were healthy individuals who had no history of either diabetes or hypertension. Patients with brain hemorrhages or tumors, patients with autoimmune or vascular disorders, and having a history of drug abuse were excluded from the study.

### 2.3 Imaging protocol

Fazekas grading is a system used to classify the severity of white matter hyperintensities (WMH) in cerebral small vessel disease (cSVD). A standard Non-Contrast Brain MRI protocol, including T1-Weighted, T2-Weighted, Fluid-Attenuated Inversion Recovery (FLAIR), Diffusion-Weighted and Susceptibility-Weighted Imaging, was used on the 1.5 T MRI machine. The axial sections of T1W, T2W, and FLAIR imaging were included only for convenience.

### 2.4 Dataset

**Fig. 1:**
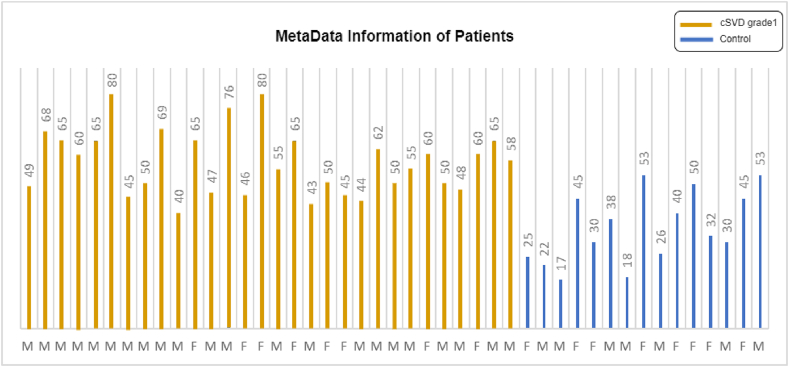
Visual representation of age & gender for patients with cSVD and controls.

The dataset contained binary classes, and highly qualified neurologists categorized the ground truth. We collected 45 patients' MRI data in the form of DICOM files containing binary classes. There were 29 male and 16 female patients. The youngest patient in the dataset was 17 years old, and the oldest was 80 years old. According to population data, the mean age of all patients was 49.8 ± 15.377 years. The DICOM files included information regarding the patient's age & gender, brain region, image type, pixel values, etc. In the dataset, binary classes are named diseased (cSVD grade-1) and normal (control). There were 15 samples from the control group and 30 samples from the diseased group within the dataset. Visual meta-information about the dataset is described in Fig. 1.

**Fig. 2:**
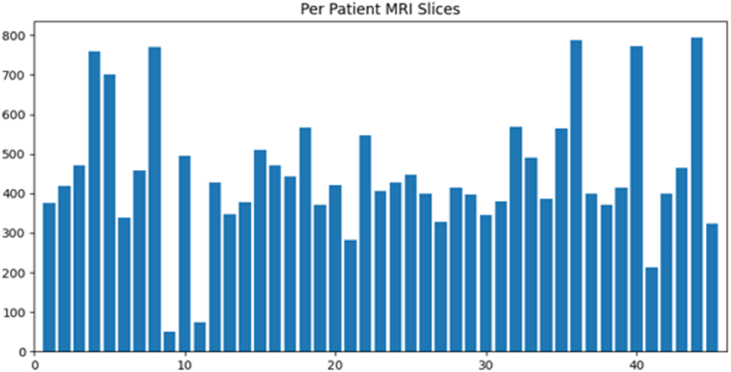
All MRI slices of each patient.

**Fig. 3:**
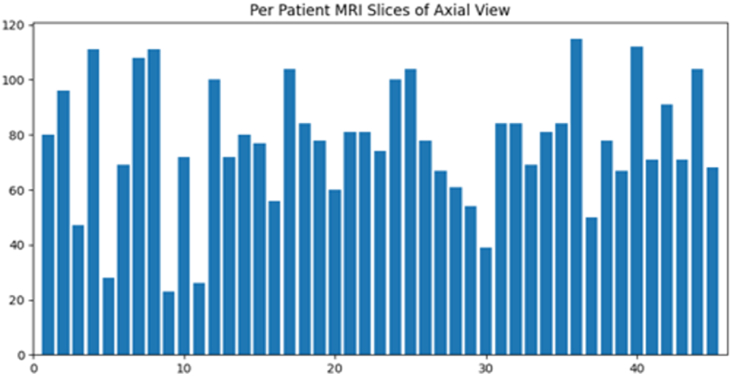
Axial view MRI slices of each patient.

The dataset was in the form of slices. Each patient's slices varied; the distance from other instance slices was 5 mm. There were axial, sagittal, and coronal sections of the MRI sequences; only the axial slices of T1W, T2W, and FLAIR imaging were included. The data of each patient's MRI slices count with all views and axial view filtered slices are in Fig. 2, Fig. 3, respectively. As Fig. 3 demonstrates, the 3D MRI dataset slices varied widely. The minimum number of slices was 23, and the maximum was 115. To create a 3D shape, we must overcome numerous obstacles, described in the methodology diagram in the following section of this study.

### 2.5 Proposed work

**Fig. 4:**
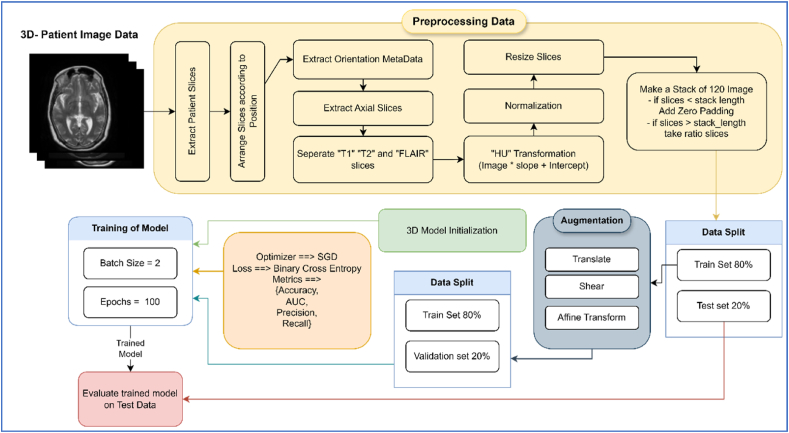
Methodology flow-diagram of proposed work.

In the proposed work, the data was pre-processed by arranging each patient's slices according to their positions and resizing each slice to make a 3D stack. Furthermore, data was normalized, and zero padding was used to make the same dimensions of a 3D stack of each patient. Adjustment of slices was the main task. Two major problems were tackled; the first was related to the imbalance dataset and the second was to make an equal number of slices of each patient. The methodology of the presented work is visually represented in Fig. 4. Each step is defined in the following subsection to show information about the pre-processing work and network architecture details.

### 2.6 Adjustment of slice positions

Each MRI slice was a DICOM file containing the metadata information except sensitive information about the patient. The meta-data had an attribute, “instance number,” and the slices were sorted according to that.

### 2.7 Data normalization

Each slice was normalized on the overall mean and standard deviation of the dataset. The data were normalized according to (1).

(1) $$\hat{x}=\frac{x-\;\mu }{\sigma }$$

The objective of normalization was to transform features into those with comparable scales. This enhanced the model's performance and training stability.

### 2.8 Resizing slices

The pixel data of each slice had a different dimension. For instance, the initial slices contained image shapes of (64, 64), while some slices also contained sizes of (256, 256) and (512,512) pixels. The images with a size of 64 were discarded because these pixels did not contain relevant information for the study in question. The remaining slices were shrunk to (128, 128) and ended with channel information. The concluding form of the single slice was (128, 128, 1).

### 2.9 Adjustment of slices

**Fig. 5:**
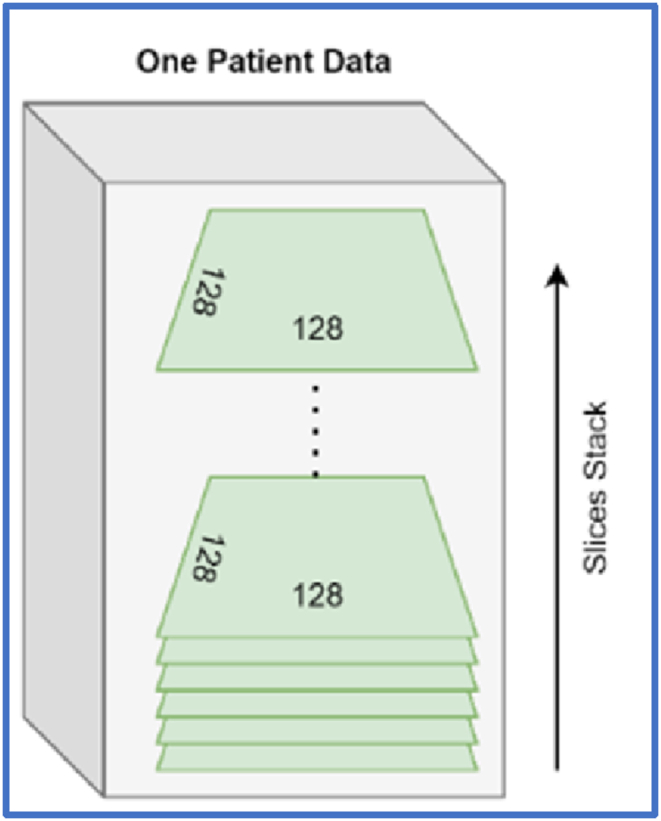
Visual representation of stack.

At the initial stage, we assumed all slices were part of the diagnosis process, so we initialized the data together to make a stack and executed processing on the data. The slice count of each patient's data in the form of a bar chart is shown in Fig. 2. As there are a maximum of more than 800 slices and a minimum of 200 slices, we experimented with different data slices, such as selecting the initial 200 slices of each patient and making a stack (47, 200, 256, 256, 1). The general view of the 3D stack is depicted in Fig. 5. We used different dimensions for 200 slices of a single patient, having a height and width of 256 with a grayscale channel.

This approach's results were accurate, but the data was too large to skip the other slices. T1, T2, and FLAIR slices of the axial view were included. Overall, the filtered slice data were significantly smaller than the slice data, and the minimum and maximum slices of the filter data were 23 and 115, respectively. In addition, the stack with different slices was managed by padding it with zero slices of the same shape to total 120 slices.

### 2.10 Zero-padding technique

A single patient's minimum and maximum slices were 23 and 115, respectively. Zero-padding was used to produce a 3D constant shape. We had a stack of 47, 120, 128, 128, 1. We experimented with this processed data; the results are represented in Section 3.

### 2.11 Splitting of data

The dataset was divided into three sections: train, test, and validation sets. First, 80 % of the data was allocated for training the model, while the remaining 20 % was reserved for testing/evaluation. In the second step, the data was augmented using the augmentation techniques of translation, shear effect, and affine transform and then again split into 80 % training data and 20 % validation data, as depicted in Fig. 4. Two augmentations of translation and affine were applied to the normal class, and the shear effect was applied to both classes. This approach ensured a robust validation strategy, enhancing the reliability of our results. We employed accuracy, AUC, recall, and precision as validation metrics to benchmark the model's performance accurately.

### 2.12 Network architecture

**Fig. 6:**
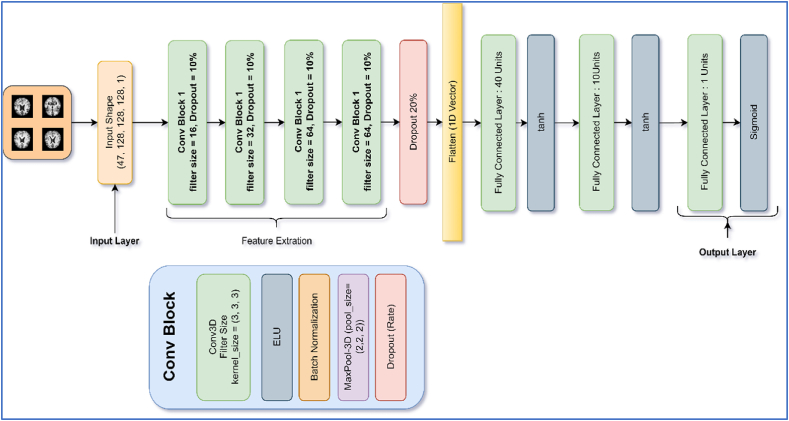
Architecture diagram of proposed work.

The main components used in the architecture are discussed in the subsections. The model architecture contained a 3D convolutional layer followed by a Batch-Normalization and Max-Pooling layer. Fig. 6 represents the visual representation of the model.

#### 2.12.1 Architecture block

As shown in Fig. 6, the network contained Conv Block followed by a flattened layer, which was used to make more than 1-D space to 1-D shape vector so that the data should feed into the fully connected (FC) layers. In addition, each layer's weights were initialized using the “He-Initialization” method. In He-initialization, the weights of a layer were initialized with random values drawn from a Gaussian distribution with a mean of 0 and a standard deviation of $\surd \frac{2}{n}$ (where n is the number of inputs to the layer). This helped to prevent the vanishing gradient problem during training. Equation (2) represents the mathematical form of He-Initialization.

(2) $$W_{i}{,}_{j}\;N(0,\sqrt{\frac{2}{n_{in}}}$$

In addition, 0.05 L2 regularization was added to each convolutional and FC layer. The summary of the model layers and trainable/non-trainable parameters is shown in Table 1.

**Table 1:** Summary of the proposed model.

Layer	Input Shape	Output Shape	Parameters
Input Layer	(None, 120, 128, 128, 1)	(None, 120, 128, 128, 1)	0
Conv Block1	(None, 120, 128, 128, 1)	(None, 59, 63, 63, 8)	256
Conv Block2	(None, 59, 63, 63, 8)	(None, 28, 30, 30, 16)	3536
Conv Block3	(None, 28, 30, 30, 16)	(None, 13, 14, 14, 32)	13984
Conv Block4	(None, 13, 14, 14, 32)	(None, 5, 6, 6, 64)	55616
Flatten	(None, 5, 6, 6, 64)	(None, 11520)	0
Fully Connected	(None, 11520)	(None, 40)	460840
Dropout	(None, 40)	(None, 40)	0
Fully Connected	(None, 40)	(None, 10)	410
Dropout	(None, 10)	(None, 10)	0
Fully Connected	(None, 10)	(None, 1)	11
**Total Parameters**	**534,653**		
**Trainable Parameters**	**534,413**		
**Non-Trainable Parameters**	**240**		

#### 2.12.2 3D-convolutional layer

3D convolution [[35], [36], [37]], was used to extract features from the input data, and by using these, the classification of the model was achieved. The layer then performed a forward pass to calculate the loss and a backward pass to update the layer's weight. Equation (3) represents the forward pass.

1. Forward Pass [35].

(3) $$y_{ijk}=\sum_{m=1}^{M}\sum_{n=1}^{N}\sum_{p=1}^{P}\left(X_{i+m-1,j+n-1,k+p-1}W_{mnp}\right)+b$$

where, x is the input tensor, w is the filter, b is the bias, M, N, and P are the dimensions of the filter, and y is the output tensor.

2. Backward Pass [35].

The mathematics of backward pass are represented in (4)–(6).

(4) $$\frac{\delta L}{\delta w_{mnp}}=\sum_{i=1}^{H}\sum_{j=1}^{W}\sum_{k=1}^{D}\left(X_{i+m-1,j+n-1,k+p-1}\left(\frac{\delta L}{\delta y_{ijk}}\right)\right)$$

(5) $$\frac{\delta L}{\delta x_{ijk}}=\sum_{m=1}^{M}\sum_{n=1}^{N}\sum_{p=1}^{P}w_{mnp}\left(\frac{\delta L}{\delta y_{i-m+1,j-n+1,k-p+1}}\right)$$

(6) $$\frac{\delta L}{\delta b}=\sum_{i=1}^{H}\sum_{j=1}^{W}\sum_{k=1}^{D}\left(\frac{\delta L}{\delta y_{ijk}}\right)$$

where L is the loss function, and $\frac{\delta L}{\delta w_{mnp}},\frac{\delta L}{\delta x_{ijk}},and\frac{\delta L}{\delta b}$ are the gradients of the loss concerning the filter, input tensor, and bias, respectively. H, W, and D are the dimensions of the input tensor.

#### 2.12.3 Activation functions

In this proposed work, we used the ELU (Exponential Linear Unit) and Sigmoid activation functions. ELU was used in the hidden layers, and sigmoid activation was used in the last (output) layer. Equations (7), (8) were used to calculate the ELU [38] and Sigmoid [39], respectively.

(7) $$f\left(x\right)=\frac{1}{1+e^{-x}}$$

(8) $$f\left(x\right)=\left\{ \begin{array}{c} x,if\;x>0\\ \alpha \left(e^{x}-1\right),Otherwise \end{array}\right.$$

#### 2.12.4 Batch-normalization

The batch normalization [40] layer was used in the network. This layer was used in each convolutional block, as represented in Fig. 6, which proved helpful in achieving the results. The batch normalization operation can be mathematically expressed in (9).

(9) $$BatchNorm\;\left(x\right)=\gamma \left(\frac{x-\mu }{\sqrt{\sigma^{2}+\epsilon }}\;\right)+\beta$$

where x is the input to the Batch Normalization layer, μ and σ are the mean and standard deviation of the current mini-batch, γ, and β are learned scaling and shifting parameters, and ϵ is a small constant added to the denominator for numerical stability.

#### 2.12.5 Max-pooling-3D

After each convolutional layer, MaxPooling 3D [37] was applied to make the model's features invariant and computationally efficient. This helped to improve the spatial resolution of the feature maps generated by the previous convolutional layer. A sliding window with dimensions of (2 × 2 × 2) was utilized. Each element in the output feature map corresponds to the maximum value of a window in the input feature map.

#### 2.12.6 Dropout layer

Dropout [41] was also utilized in the model architectures before converting it into a 1D vector. This was helpful to prevent the model from overfitting in the training process. A dropout was used to randomly off the 20 % of neurons, forcing the remaining neurons to acquire more robust features. The model was overfitted in experimentation with different steps, but this issue was overcome by reducing convolutional and dropout layers in the architecture.

#### 2.12.7 Fully connected layer

In a neural network, a fully connected layer in which all nodes from the previous layer are connected to all nodes in the current layer [42]. After performing a linear operation on the input, this layer applies an activation function to the output. The calculations for an FC [29] layer are shown in (10).

(10) y=f(∑Wx+b)

where x defines the input vector, W represents the weight matrix, b reflects the bias vector, f denotes the activation function, and y indicates the output vector.

### 2.13 Evaluation metrics

The proposed model was evaluated using different metrics. We employed different metrics to benchmark our model's performance. Four terms: accuracy, the area under the curve, recall, and precision are extensively used when observing various metrics of a classifier.

#### 2.13.1 Accuracy

In this study, binary classes were utilized for classification, and binary accuracy was applied to evaluate the model's accuracy. The mathematical representation of accuracy [42] metric is given in (11).

(11) $$Accuracy=\frac{TP+TN}{TP+TN+FP+FN}$$

where TP, FP, TN, and FN stand for True Positive, False Positive, True Negative, and False Negative, respectively.

#### 2.13.2 Area under curve

The area Under Curve (AUC) evaluation metric was also used to measure the model's performance. The mathematical representation of AUC [43] is given in (12).

(12) $$AUC=\int_{0}^{1}TPR\;\left(FPR^{-1}\left(t\right)\right)dt$$

Where TPR is the true positive rate, FPR is the false positive rate, and $FPR^{-1}\left(t\right)$ is the inverse function of the FPR at threshold t. The AUC value ranges between 0 and 1, with higher values indicating better performance.

#### 2.13.3 Recall

This work also used recall as an evaluation metric with other metrics. This is also known as sensitivity or true positive rate and was used to examine the model's ability to identify positive samples correctly. It is the ratio of true positive predictions to the total number of positive samples in the dataset. The mathematical representation for recall [29,33] is given in (13).

(13) $$Recall=\frac{TruePositive}{TruePositive+FalseNegative}$$

#### 2.13.4 Precision

Precision or specificity was calculated to measure the proportion of true positive predictions made by the model out of all positive predictions. The mathematical representation for precision [29,33] is given in (14).

(14) $$Precision=\frac{TruePositive}{TruePositive+FalsePositive}$$

#### 2.13.5 Binary cross-entropy loss

Binary Cross-Entropy (BCE) Loss was calculated to predict the difference between the predicted and actual output of the model. The formula for binary cross-entropy [44] loss is depicted in (15).

(15) $$L\left(y,\hat{y}\right)=-\frac{1}{N}\sum_{i=1}^{N}y_{i}\;\log\left({\hat{y}}_{i}\right)+\left(1-y_{i}\right)\log\;\left(1-{\hat{y}}_{i}\right)$$

where y is the true label (either 0 or 1), ˆy is the predicted probability (ranging from 0 to 1), and N is the number of samples in the dataset.

## 3 Experimentation & results

The experiments of this study were performed on Kaggle, with the specification of 16 GB RAM, 16 GB Graphical Processing Unit (GPU) NVIDIA QUADRO P100, and 70 GB Hard Disk. The model was evaluated using the test set produced from splitting the dataset before augmentation and training the model. Utilizing multiple metrics guarantees a model's robustness from every perspective. The combined comprehension of these results defines a model's effective training. There are also additional elements at work, like loss, overfitting, and so forth.

### 3.1 Experimentation on 2D dataset

Two experiments were conducted with both all slices and only axial slices. Both topics were discussed in the subsections.

#### 3.1.1 Experimentation on 2D all slices

Each patient's data contained a different number of MRIs, and the dataset has been pre-processed. The dataset was initially converted to 2D images, and the two disease directories contained a total of 20173 samples, including 6174 images in the control class and 13999 as diseased samples. Experimentation with 2D models was conducted using pre-trained models for feature extraction, followed by the addition of fully connected layers and the ReLU activation function. The last layer contained the Sigmoid activation function with one neuron as we worked on the binary classification. The loss was calculated using the formula of binary cross-entropy loss, and the accuracy matrices were used as binary accuracy, precision, recall, and AUC. The experimentation with various optimizer learning rates (LR) was repeated multiple times, and the optimal LR was 0.001. The LR in training was then decreased using the ReduceLROnPleateu callback. Different pre-trained models' experiments are summarized in Table 2.

**Table 2:** Transfer learning on 2D images with all samples.

Sr. No	Model Name	Dropout	Optimizer	Loss	Acc%	AUC%	Precision%	Recall%
1	MobileNet-V2	–	SGD (LR 0.1)	0.7009	65.31	69.43	74.045	70.685
2	VGG19	–	SGD (LR 0.001)	2.882	56.78	70.23	78.71	44.5684
3	VGG16	20 %	SGD (LR 0.001)	0.3023	52.67	73.34	73.34	41.145
4	DenseNet121	–	SGD (LR 0.01)	0.7275	65.5	75.34	75.34	60.79
5	ResNet50-V2	–	SGD (LR 0.01)	0.6646	64.17	64.15	64.15	99.99

#### 3.1.2 Experimentation on 2D axial slices

The procedure of transfer learning was performed on the 2D Axial slices. These axial slices were converted into 2D shapes and the slices were used as input in the 2D model. Each slice was normalized such that the mean was equal to 0 and the standard deviation was equal to a unit. After converting only axial slices, there were 2512 samples in the diseased set and 1043 samples in the normal set. Table 3 represents the experimentation completed on the dataset.

**Table 3:** Transfer learning on 2D axial slices.

Sr. No	Model Name	Dropout	Optimizer	Loss	Acc%	AUC%	Precision%	Recall
**1**	**VGG16**	**20 %**	**SGD (LR 0.001)**	**0.1639**	**93.12**	**98**	**93.26**	**83.84**
2	DenseNet121	20 %	Adam (LR 0.0001)	0.1861	93.12	96.98	89.19	82.50
3	VGG19	–	Adam (LR 0.001)	0.2439	91.25	97.10	89.39	73.75
4	ResNet50-V2	20 %	Adam (LR 0.001)	0.2139	91.56	96.77	94.81	76.04
**5**	MobileNet-V2	20 %	Adam (LR 0.0001)	0.2211	92.81	96.74	91.95	83.33

Table 3 provides the results of an experiment conducted with five different pre-trained models on a dataset. The Adam optimizer was used to optimize the models' performance in all experiments except Experiment 1 (VGG16), and a dropout rate of 20 % was employed in all experiments until Experiment 3 (VGG19). In Experiment 1, we employed the VGG16 model; this experiment yielded a loss value of 0.1639, an accuracy of 93.12 %, an AUC score of 98 %, a precision of 93.26 %, and a recall of 83.84 %. Moving on to Experiment 2, the DenseNet121 resulted in a loss value of 0.1861, an accuracy of 93.12 %, an AUC score of 96.98 %, a precision of 89.19 %, and a recall of 82.5 %. Experiment 3 employed the VGG19 model without dropout, utilizing the Adam optimizer. This experiment yielded a loss value of 0.2439, an accuracy of 91.25 %, an AUC score of 97.1 %, a precision of 89.39 %, and a recall of 73.75 %.

In Experiment 4, the ResNet50-V2 model achieved a loss of 0.2139, an accuracy of 91.56 %, an AUC score of 96.77 %, a precision of 94.81 %, and a recall of 76.04 %. Finally, Experiment 5 employed the MobileNet-V2 model with a 20 % dropout rate, utilizing the Adam optimizer. This configuration achieved a loss value of 0.2211, an accuracy of 92.81 %, an AUC score of 96.74 %, a precision of 91.95 %, and a recall of 83.33 %.

Among all the experiments, the VGG16 model (Experiment 1) exhibited the highest accuracy of 93.12 %, making it a notable configuration in terms of classification performance.

### 3.2 Comparison with 3D-models

The model's architecture, as proposed in Fig. 6, had been selected after multiple experiments on the model. At each level, various pre-processing techniques and hyper-parameter adjustments were implemented. The proposed model was trained through the addition of L2 regularization, weight initialization of each layer, dropout layers, and FC layers. Table 4 shows the comparison results with different models and pre-processing techniques. The hardware was also the focus of our investigation. A single image stack was too large to be fed into the Graphical Processing Unit (GPU) and used to train a model. The experiments on the dataset were performed by implementing the pre-processing steps in the proposed section. There were very few training parameters in the model. Further, there were two sections categorized from which we evaluated our model performance on the axial slices and by taking random slices of a single patient.

**Table 4:** Proposed 3D model test results.

Sr. No	Conv. Layers	Trainable Parameters	Optimizer	Aug.	Loss	Acc%	AUC%	Precision%	Recall%
1	Six	3.086 M	SGD	None	0.4270	75	80	77	73
2	Five	2.75 M	SGD	R, T, S, AT	0.6745	73.33	72.7	76.92	90.9
3	Five	1.245 M	SGD	R	0.4906	80	78.41	78.57	99.99
4	Five	1.245 M	Adam	T	0.5742	73.33	65.91	76.92	90.91
**5**	**Four**	**0.534 M**	**Adam**	**R, AT**	**0.3412**	**85.71**	**86.25**	**83.33**	**99.99**

Key: R for rotation, T for Translation, S for shear effect, and AT stands for Affine Transform.

SGD: stochastic gradient descent.

### 3.3 Axial slices 3D experimentation

Table 4 shows the performance of the tests that have been conducted. Each test experimentation contained a different architecture or change in the optimizer. The “Conv. Layer” column shows information about how many convolutional layers were in the model architecture, and the “Aug” column shows which augmentation was used. Further columns represent the testing values of the model's loss, accuracy, AUC, precision, and recall scores.

The 3D experimentation on the model is mentioned in Table 4. Furthermore, we compared the last highest accuracy 3D model with and without augmentation with K-Fold Cross-validation. While experimenting without augmentation, we took the testing ratio of 90 % train and 10 % test set. We applied 6-fold Cross-validation on the train set to obtain the validation data from the train set and test on the before split set. On the other hand, with augmentation, the ratio of the train and test set was the same, and did it before augmentation. In augmentation, we applied rotation on both classes and affine transform on the normal class to balance the dataset samples. After augmentation, 5-fold Cross-validation was applied with a stratified split to the dataset. Comparisons of accuracy, AUC, precision, recall, and loss are mentioned in the next subsections.

Table 4 presents the results of several network experiments with varying configurations. Experiment 1, with six convolutional layers and 3.086 million trainable parameters, achieved a loss of 0.4270, an accuracy of 75 %, an AUC score of 80 %, a precision of 77 %, and a recall of 73 %. In Experiment 2, with five convolutional layers and 2.75 million trainable parameters, using the SGD optimizer with additional augmentations (R, T, S, AT), the model obtained a loss of 0.6745, an accuracy of 73.33 %, an AUC score of 72.7 %, a precision of 76.92 %, and a recall of 90.9 %.

Experiment 3, also with five convolutional layers but only 1.245 million trainable parameters, utilized the SGD optimizer with augmentation (R) and resulted in a loss of 0.4906, an accuracy of 80 %, an AUC score of 78.41 %, a precision of 78.57 %, and a recall of 99.99 %. In Experiment 4, the same configuration as Experiment 3 was used, but with the Adam optimizer and no augmentation (T). This experiment yielded a loss of 0.5742, an accuracy of 73.33 %, an AUC score of 65.91 %, a precision of 76.92 %, and a recall of 90.91 %.

Finally, Experiment 5 employed four convolutional layers with 0.534 million trainable parameters, utilizing the Adam optimizer with augmentations (R, AT). This configuration achieved a loss of 0.3412, an accuracy of 85.71 %, an AUC score of 86.25 %, a precision of 83.33 %, and a recall of 99.99 %. These experimental results provide insights into the performance of different network configurations and their respective evaluation metrics. Among all the experiments conducted, this particular configuration showcased the highest accuracy of 85.71 %. This noteworthy result highlights the effectiveness of this specific 3D model in achieving superior classification performance.

### 3.4 Accuracy comparison

**Fig. 7:**
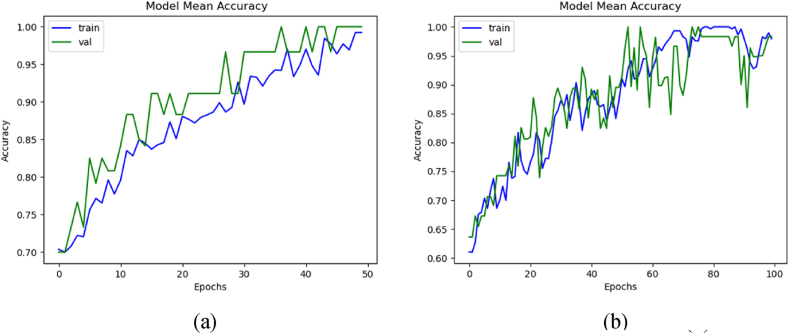
Mean accuracies of the 3D-models: (a) with data augmentation, and (b) without data augmentation.

The accuracy comparison with and without dataset augmentation was calculated using the formulas given in Section 2. Without augmentation, the graph shows straight horizontal lines because of insufficient data. These graphs were taken using the mean values of all the K steps. Fig. 7(a) displays the mean accuracy of training and validation with augmentation, while Fig. 7(b) demonstrates the accuracy without augmentation. The model trained with augmented data achieved the mean training, validation, and testing accuracies of 97.95 %, 98.09 %, and 85.71 %, respectively. Whereas without augmentation, it secured the mean training, validation, and testing accuracies of 98.89 %, 99.99 %, and 75 %, respectively.

### 3.5 AUC comparison

**Fig. 8:**
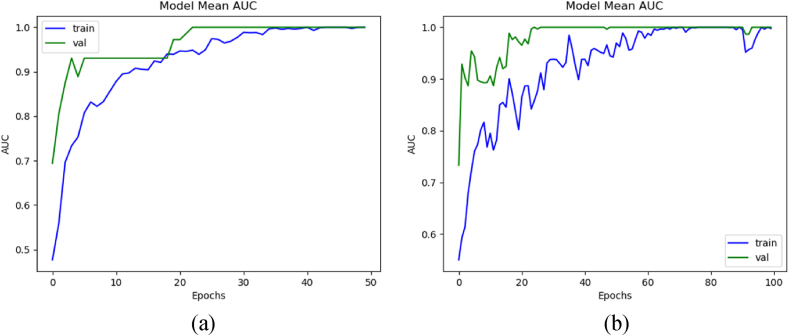
Mean AUC of the 3D-models: (a) with data augmentation, and (b) without data augmentation.

The AUC was compared with and without dataset augmentation using the formulae given in Section 2. These graphs were taken using the mean values of all the K steps. Fig. 8(a) depicts the mean AUC of train and validation with augmentation, whereas Fig. 8(b) depicts the AUC without augmentation. The model trained with augmented data achieved the mean training, validation, and testing AUC of 99.95 %, 99.99 %, and 86.25 %, respectively. Whereas without augmentation, it secured mean training, validation, and testing AUC of 98.89 %, 99.99 %, and 80 %, respectively.

### 3.6 Recall comparison

**Fig. 9:**
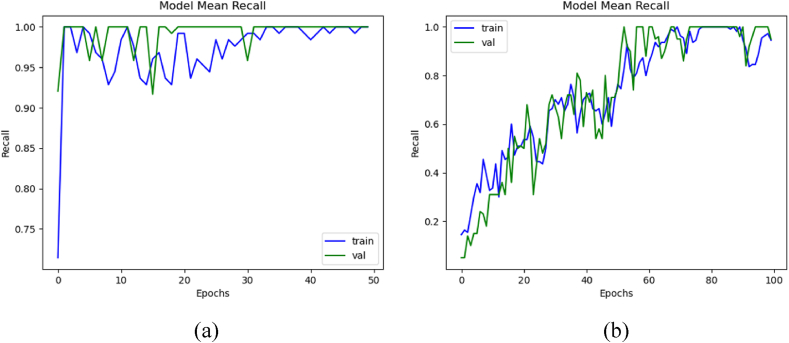
Mean recall of the 3D-models: (a) with data augmentation, and (b) without data augmentation.

Comparison with and without augmentation with K-Fold validation steps was evaluated. The average scores among all K steps were employed to generate graphs. Fig. 9(a) shows the mean recall of training and validation with augmentation, whereas Fig. 9(b) illustrates the recall without augmentation. The model trained with augmented data achieved the mean training, validation, and testing recall of 93.81 %, 94.15 %, and 99.99 %, respectively. Whereas without augmentation, it secured mean training, validation, and testing recall of 99.99 %, 99.99 %, and 73 %, respectively.

### 3.7 Precision comparison

**Fig. 10:**
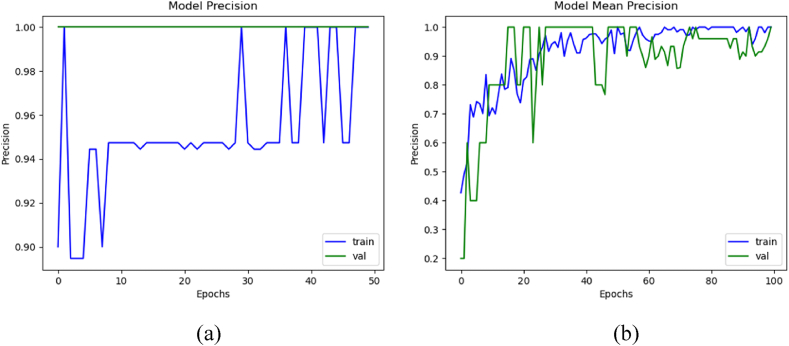
Mean precision of the 3D-models: (a) with data augmentation, and (b) without data augmentation.

Using formulas given in Section 2, the precision evaluation between each dataset augmentation. The average scores among all K steps were employed to generate these graphs. Fig. 10(a) shows the mean precision of training and validation with augmentation, whereas Fig. 10(b) illustrates the precision without augmentation. The model trained with augmented data achieved the mean training, validation, and testing precision of 99.95 %, 99.90 %, and 83.33 %, respectively. It secured mean training, validation, and testing precision of 99.99 %, 99.99 %, and 77 %, respectively, without augmentation.

### 3.8 Loss comparison

**Fig. 11:**
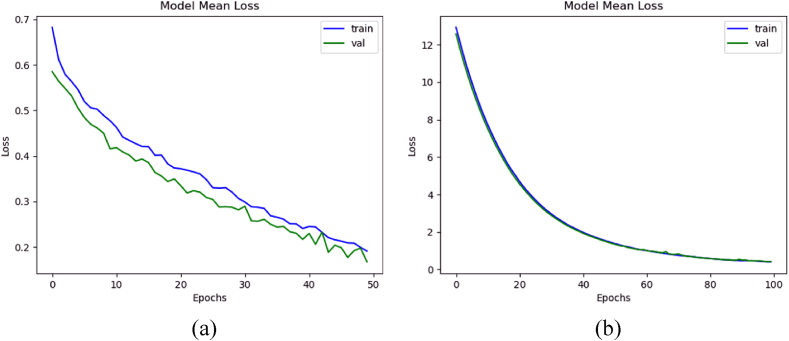
Mean loss of the 3D-models: (a) with data augmentation, and (b) without data augmentation.

The loss comparison with and without dataset augmentation was calculated using the formulas given in Section 2. These graphs were taken using mean loss values of all the K steps. Fig. 11(a) displays the mean training and validation loss with and without augmentation, while Fig. 11(b) shows the loss when trained with data augmentation. The model trained with augmented data achieved the mean training, validation, and testing loss with augmentation were 0.1256, 0.1215, and 0.3412, respectively. Whereas when trained without data augmentation, it secured mean training, validation, and testing loss of 0.19, 0.17, and 0.4270, respectively.

## 4 Discussion

Machine learning algorithms and artificial intelligence systems have shown great promise in a variety of fields of healthcare, including precise illness grade diagnosis, patient outcome prediction, and picture interpretation automation. In this study, we developed an AI-aided diagnostic system with improved accuracy for the earliest detection of grade-1 cSVD. Specifically, we were interested in extracting and identifying AI-based markers and features for cSVD from locally collected MRI data using advanced deep-learning techniques. There are numerous classification algorithms for 3D medical images on cSVD. Despite advances in neuroimaging and biomarkers in recent decades, the pathophysiology of vascular disease remains unknown. In the current study, the VGG16 model had the highest accuracy of 93.12 % across all trials on the 2D axial slices, making it a significant configuration in terms of classification performance. On 3D-axial slices, the experiment with four convolutional layers, 0.534 million trainable parameters, and the Adam optimizer with augmentations demonstrated a maximum accuracy of 85.71 %. This notable result reflects the efficiency of this particular 3D model in obtaining improved classification performance.

A review discussed the function of deep machine techniques and other aspects of cSVD by tying it to many cerebral and non-cerebral disorders and current breakthroughs in the area to achieve a sensitive diagnosis, effective prevention, and disease treatment. Because medical imaging plays a crucial role in diagnosing cSVD, interest in using computerized techniques, notably neural networking, machine learning, and deep learning in image processing, has grown dramatically. Recently, the machine learning paradigm has been utilized to stratify stroke risk using ultrasonic echolucent carotid wall plaque morphology [45]. Another study found that embedding a polling-based principal component analysis strategy into a machine learning framework to select and retain dominant features improved the performance of stroke risk stratification based on plaque tissue morphology using carotid ultrasound [46] and Gaussian Process Regression (GPR) was utilized [47] in combination with a novel automated white matter lesion segmentation method and lesion healing step to determine the severity of SVD. WMH altered the volume of white matter, which was quantified and utilized as a covariate of interest in voxel-based morphometry and voxel-based cortical thickness analyses. Up to 80 % accuracy has been obtained in diagnosing cSVD by trained support vector machines and radial basis functions using DTI-derived features [48]. A semi-automated residual 3D Convolutional Neural Network (CNN) has been utilized to classify magnetic resonance (MR) scans [28]. Researchers perform well on the custom dataset, but they classify the T1 and FLAIR portions independently. A large dataset has been used to test the proposed designs for segmenting white matter hyperintensities in brain MR images [34]. Consequently, it was discovered that CNNs that contain location information outperform a traditional segmentation technique using handmade characteristics and CNNs that do not incorporate location information. The best configuration of their networks had a Dice score of 0.792 on a test set of 50 scans, compared to 0.805 for an independent human observer. However, they discovered that the machine's and the independent human observer's performance levels were not statistically substantially different. Researchers have presented a two-stage automated technique based on deep convolutional neural networks (CNN). They demonstrated that this strategy performs well and can significantly assist readers [20]. We begin by detecting initial candidates using a fully convolutional neural network. The resultant CAD system performs similarly to trained human observers, with a sensitivity of 0.974 and 0.13 false positives per slice. A feasibility study also showed that a trained human observer would greatly benefit from the CAD system's assistance. An explainable deep learning has been used to detect brain hemorrhages and their locations in scans. Normal brain scans with no hemorrhages were compared to computed tomography images with subarachnoid, intraventricular, subdural, epidural, and intraparenchymal hemorrhages. A ResNet deep learning model with image processing was used. The system performed well, with an accuracy of 0.81, sensitivity of 0.67, and specificity of 0.86. These findings provide proof of concept for the application of explainable artificial intelligence (XAI) to detect and visualize brain hemorrhages in medical pictures, allowing for faster diagnosis and treatment [30].

AddNet classifier has also been designed to classify MR images with dementia on multiple scales and to deal with data imbalance. It was intended to create an optimized network; however, their method incorporated the synthetic samples into the test data [29]. The MRIs have been used to establish a reliable and efficient approach for detecting Alzheimer's disease using a deep Convolutional Neural Network (CNN). They presented a CNN architecture for detecting Alzheimer's disease with limited parameters, making it ideal for training a smaller dataset. This suggested approach distinguishes between the early stages of Alzheimer's disease and presents class activation patterns on the brain as a heat map [31]. There is a lot of evidence that cSVD has a role in the incidence of AD. It has been well established that there is a link between Alzheimer's disease and major imaging indicators of cSVD, such as lacunar infarction, WMH, and CMBs (cerebral microbleeds) [49]. A computer-aided diagnostic system has been developed based on minor blood vessel abnormalities in MRI images, employing CNN and deep learning methods to assess brain vascular occlusion via brain MRI images. They examined MRI images from 50 patients [50]. This technology primarily assists clinicians in determining if cerebral small vessel lesions exist in brain MRI scans and converting the discovered data into labeled pictures. Finally, all of the patient's MRI images are synthesized, displaying a 3D representation of the tiny blood vessels in the brain to aid the doctor in reaching a diagnosis or providing the correct lesion location for the patient.

## 5 Visualization through gradient-weighted class activation map

**Fig. 12:**
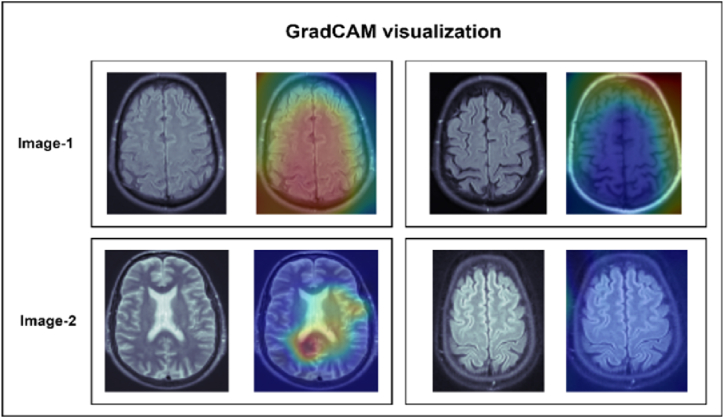
Generalization of the class activation map through GradCAM.

Grad-CAM (Gradient-weighted Class Activation Mapping) [51] identifies the regions most important for CNN classification by calculating a Class Activation Map (CAM) using gradient information. Grad-CAM generates a visual map highlighting the areas in the input image most influential for the model's predictions. This technique helps understand the network's perception and which neurons are activated in specific deep layers. To generate the CAM, the gradient of the class score is computed with respect to the feature maps of the CNN layers. The resulting gradients are then used to weight the feature maps, producing a localization map that shows the critical regions affecting the model's prediction. This process ensures that the final CNN layer generates a CAM that highlights the image areas most relevant to the classification task, as illustrated in Fig. 12.

The Grad-CAM algorithm was employed to identify the features most important in the model's predictions to ensure model interpretability. Visualizations were created to show the impact of different regions on the predicted outcomes, enhancing the understanding of the model's decision-making process. In our study, each 3D image contained 120 slices, and two slices from each class are shown in Fig. 12. The highlighted regions in these slices indicate the model's key features. Notably, the second slice of both classes did not highlight any regions, as they did not contain significant information related to the disease. These visualizations demonstrate the model's interpretability regarding feature selection.

## 6 Conclusion & recommendation

The proposed work evaluated a novel approach to cSVD grade-1 detection using a lightweight deep-learning model. Overall, the 3D-CNN model performed very well on the test set, with an accuracy of 87.5 % on 3D axial slices. Computational imaging technologies that provide the earliest and most accurate diagnosis can help minimize the burden of cSVD and its related morbidity, such as vascular dementia. With the earliest detection, we can reduce the public's burden of neurological disorders, allowing sufferers to live more productive lives. In the future, we will try to deploy the trained model at the collaborative hospital(s). We intended to implement this model as a computer-aided diagnostic system for the early diagnosis of grade-1 cSVD. However, practical implementation and integration into clinical workflows require further exploration. A feasibility study is needed to assess the system's deployment in real-world settings, considering factors like cost-effectiveness and interoperability with existing medical systems. Future research should also explore training clinicians, data privacy concerns, and compliance with healthcare regulations to ensure smooth integration and practical utility.

## 7 Limitations

This pilot study was conducted in a single clinical setting, which may limit its applicability to other populations or healthcare settings. In addition, the dataset was imbalanced, which could affect the model's performance and reliability. These limitations should be considered when interpreting the results and generalizing the study's findings. To validate and extend the current findings, it would be beneficial to conduct additional research with larger and more balanced datasets and in various clinical settings.

## Ethical approval

The study conformed to institutional ethical standards. A prior ethical approval (ref #: 145/NEU-MH/2023) on January 23, 2023 was taken from the Neurology Department, King Edward Medical University (KEMU), Mayo Hospital, Lahore, Pakistan.

## Informed consent

The participants gave verbal informed consent because they were not directly involved in the study. Only their basic demographics (age, gender) and radiological data were used. In addition to parental/guardian consent, consent was also obtained from minors.

## Consent to publish

All authors agreed to publish this article.

## Code availability statement

The code of preprocessing and modeling is available at “https://github.com/shahidzikria/Computational-imaging-for-rapid-detection-of-grade-I-cerebral-small-vessel-disease-cSVD”.

## Financial interests

We have no financial interests to declare.

## CRediT authorship contribution statement

**Saman Shahid:** Writing – review & editing, Supervision, Project administration, Funding acquisition, Conceptualization. **Aamir Wali:** Visualization, Validation, Resources, Methodology. **Sadaf Iftikhar:** Visualization, Investigation, Data curation, Conceptualization. **Suneela Shaukat:** Visualization, Software, Resources, Data curation. **Shahid Zikria:** Writing – original draft, Validation, Methodology, Formal analysis. **Jawad Rasheed:** Visualization, Methodology, Formal analysis. **Tunc Asuroglu:** Visualization, Software, Formal analysis.

## Declaration of competing interest

The authors report no conflict of interest.
